# Influence of Interfacial Stress on the Structural Characteristics and Hydrogen Sensing Performance of WO_3_ Films

**DOI:** 10.3390/nano15231785

**Published:** 2025-11-27

**Authors:** Zhihong Qiao, Jianmin Ye, Wen Ye, Jie Wei, Ying Li, Zhe Lv, Meng Zhao

**Affiliations:** 1 School of Physical Science and Technology, Suzhou University of Science and Technology, Suzhou 215000, China; 2 School of Information and Communication, Harbin Institute of Technology, Harbin 150001, China; 3 Advanced Microscopy and Instrumentation Research Center, Harbin Institute of Technology, Harbin 150080, China; 4 School of Electronic and Information Engineering, Suzhou University of Science and Technology, Suzhou 215000, China

**Keywords:** hydrogen sensor, optical and electrical responses, WO_3_ film, interfacial stress

## Abstract

Tungsten trioxide (WO_3_) exhibits complementary optical and electrical responses toward hydrogen, yet the interplay between interfacial stress, crystal phase stabilization, and gasochromic/chemiresistive performance remains insufficiently understood. In this work, WO_3_ films were grown on four single-crystal oxide substrates to systematically tune interfacial stress and thereby modulate the resulting crystal phase, microstructure, and exposed facets. θ–2θ diffraction revealed that WO_3_ adopts a monoclinic phase on YAlO_3_ and SrLaAlO_4_, whereas a high-temperature orthorhombic phase is stabilized on LaAlO_3_ (LAO) and SrTiO_3_ due to stronger interfacial constraint. Compared with the amorphous quartz reference, the single-crystal substrates significantly enhanced both gasochromic and chemiresistive responses. In particular, the orthorhombic WO_3_/LAO film exhibited an electrical response of 1.97 × 10^4^ (R_air_/R_H2_), an optical transmittance changed of 12.7%, and an electrical response time of 1 s toward 2% H_2_ at 80 °C, far exceeding the monoclinic and amorphous counterparts. The combined effects of stress-induced phase stabilization, film orientation, and hydrogen diffusion pathways are shown to govern the non-monotonic sensing trends among different substrates. These findings elucidate the structural origin of hydrogen sensitivity in WO_3_ and provide guidance for stress-engineered design of high-performance gasochromic and chemiresistive sensors.

## 1. Introduction

The hydrogen energy sector has undergone significant development [1,2], propelled by extensive governmental attention and considerable financial investment on a global scale. However, conventional hydrogen sensors have proven inadequate in meeting the rigorous demands for response time and stability required by emerging applications such as fuel cell vehicles and hydrogen refueling stations [3,4], resulting in frequent safety incidents. Consequently, the development of high-performance hydrogen sensors specifically designed to address the technical requirements of the hydrogen energy industry is of paramount importance [5,6,7]. Metal oxides (MO_x_) based hydrogen sensors exhibit high sensitivity and rapid response characteristics, and recent progress in sensor array technologies and pattern recognition algorithms has mitigated their selectivity limitations [8]. Presently, the primary technical challenges impeding their widespread application in the hydrogen energy domain include achieving a broad detection range (0.1–4%), ensuring environmental adaptability (operating effectively under pressures of 60–110 kPa, temperatures ranging from −40 to 85 °C, and humidity levels spanning 0 to 100%), and securing a long operational lifespan (≥10 years) [5].

Among various oxide semiconductors, tungsten trioxide (WO_3_) exhibits particularly versatile applicability in diverse scenarios [9,10], with an especially notable performance in hydrogen (H_2_) sensing. WO_3_ not only exhibits behavior typical of n-type oxide semiconductors—where surface reactions between hydrogen and adsorbed oxygen ions lead to a reduction in electrical resistance—but also, when combined with appropriate catalysts such as platinum (Pt) or palladium (Pd), undergoes a bulk reaction induced by the “double injection” of hydrogen species [11,12,13]. This bulk reaction results in a characteristic blue coloration of WO_3_ and a further decrease in resistance due to increased carrier concentration. Literature reviews indicate that the resistance changes in WO_3_ caused by surface adsorption reactions are sensitive to low H_2_ concentrations but tend to saturate quickly [14,15,16], whereas the hydrogen-induced colorimetric changes associated with double injection bulk reactions are more suitable for detecting higher H_2_ concentrations [17,18,19,20,21]. Current Pd/WO_3_ gasochromic and chemiresistive studies rarely meet the DOE-specified 0.1–4% H_2_ detection range and long-lifetime targets simultaneously, and systematic performance benchmarking remains limited. Therefore, elucidating the regulatory mechanisms governing WO_3_’s optical and electrical responses and developing H_2_ sensors with an extended detection range and synchronized electrical and optical response characteristics is of significant scientific and practical value.

**Figure 1:**
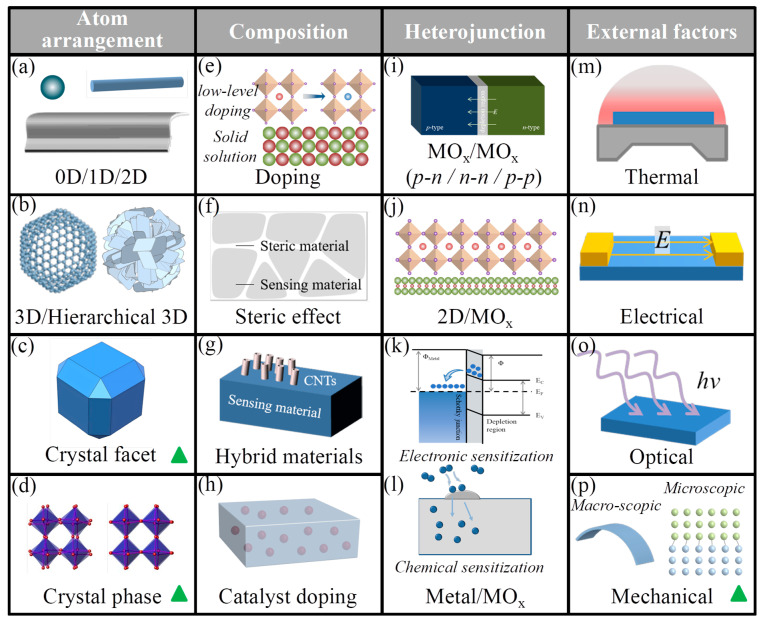
Common methodologies for adjusting the sensitivity of gas sensing materials based on MO_x_. (**a**–**d**) Atom arrangement, (**e**–**h**) compostion, (**i**–**l**) heterojunction, (**m**–**p**) external factors.

Figure 1 depicts commonly employed strategies for tuning the response performance of MO_x_ gas-sensitive materials, which can be categorized into four principal types. The first category [22,23], termed atom arrangement, primarily involves the control of morphology and structure (Figure 1a–d). Morphological regulation (Figure 1a,b) pertains to the manipulation of nanoscale building blocks of the sensing material, encompassing zero-dimensional (0D), one-dimensional (1D), two-dimensional (2D), and three-dimensional (3D) architectures. Notably, 3D hierarchical nanomaterials often comprise assemblies of lower-dimensional components, such as nanoflowers constructed from 2D nanosheets or nanospheres formed by 0D nanoparticles. The second category [24,25], composition control, includes strategies such as low-concentration substitutional doping, formation of high-concentration solid solutions, grain growth inhibition via spatial steric effects, synthesis of hybrid materials combining carbon nanotubes (CNTs) or various organic compounds with MO_x_, and homogeneous catalyst doping (Figure 1e–h). Given the critical role of heterojunctions [26,27] in modulating gas sensitivity, heterostructured layered materials are distinguished from composition control and further subdivided into MO_x_/MO_x_ heterojunctions, 2D material/MO_x_ heterojunctions, and metal material/MO_x_ heterojunctions (Figure 1i–l). When noble metal catalysts are involved, two principal mechanisms—electronic effects and catalytic effects—are operative. The fourth category encompasses external parameter control [28,29], which includes regulation of operating temperature (either constant or variable), modulation of electric field strength, light-assisted gas sensing, and mechanical force application (Figure 1m–p). Mechanical force control is further differentiated into macroscopic deformation and microscopic interfacial stress between films and substrates. Among these tuning approaches, control over crystal facets, crystal phases, and interfacial stress is relatively uncommon (highlighted by the green triangle in Figure 1c,d,p), yet these factors are intricately interrelated within the specialized framework of epitaxial films.

The crystal phase and exposed crystal facets of oxide epitaxial films can be precisely manipulated through the selection of single-crystal substrates with specific types and orientations, thereby providing ideal experimental platforms to investigate the influence of crystal phase and facet exposure on gas sensing performance [30]. Prior studies have successfully fabricated epitaxial WO_3_ thin films exhibiting single exposed crystal facets in tetragonal [31,32], monoclinic [32], and hexagonal phases [33] on various substrates. Furthermore, oxide epitaxial films offer advantages such as controllable vertical grain size, and excellent thermal stability, rendering them highly promising candidates for gas sensing applications. WO_3_ is particularly well-suited for studies on crystal phase and facet regulation due to its six distinct crystal phase transitions occurring between −100 °C and 1000 °C [34,35]: monoclinic (Pc, <−55 °C), triclinic ($\bar{P1}$, −55 to 15 °C), monoclinic (P2_1_/n, 15 to 350 °C), orthorhombic (Pmnb, 350 to 720 °C), monoclinic (P2_1_/c, 720 to 800 °C), tetragonal (P4/ncc, 800 to 900 °C), and tetragonal (P4/nmm, >900 °C). Additionally, a hexagonal phase of WO_3_ exists, and at ambient temperature, WO_3_ may exhibit coexistence of triclinic and monoclinic phases. Although the complexity of these crystal structures poses challenges for the preparation of specific phases, it simultaneously affords greater flexibility for tuning gas sensing properties. From a crystallographic perspective, the phase transitions in WO_3_ fundamentally involve distortion and tilting of WO_6_ octahedra [31]. Consequently, by selecting different single-crystal substrates, interfacial stress between the epitaxial film and substrate can be exploited to modulate the distortion and tilting of WO_6_ octahedra, enabling the stabilization of metastable phases absent at room temperature and thereby achieving novel gas sensing performance. Concurrently, the orientation of the single-crystal substrate can be utilized to control the exposed crystal facets of WO_3_ films. In this study, laser molecular beam epitaxy (Laser-MBE) is employed to synthesize WO_3_ thin films with tailored crystal phases and exposed facets on single-crystal substrates of varying orientations and lattice constants, with the objective of elucidating the effects of crystal phase and facet exposure on the optical and electrical hydrogen sensing properties of WO_3_ [36].

## 2. Experimental Section

### 2.1. Selection of Substrate

From a structural standpoint, WO_3_ can be conceptualized as a perovskite oxide (ABO_3_-type structure) lacking the A-site cation [31,34]. The absence of this A-site cation permits the oxygen octahedra within WO_3_ to undergo significant tilting and twisting. Consequently, variations in temperature, the incorporation of ions such as H^+^ and Li^+^, or the deposition of WO_3_ onto different single-crystal substrates induce modifications in its crystal structure, including changes in lattice parameters and crystal symmetry [12,13,37]. Table 1 summarizes the prevalent WO_3_ crystal phases alongside their respective lattice constants. Alterations in the WO_3_ crystal structure and the orientation of exposed crystal planes are known to influence its gas sensing properties. Guided by this hypothesis and adhering to the criterion of selecting substrates exhibiting lattice mismatches below 10%, four widely utilized single-crystal oxide substrates were chosen: YAlO_3_ (YAO), SrLaAlO_4_ (SLAO), LaAlO_3_ (LAO), and SrTiO_3_ (STO) (MTI, Hefei, China). The lattice constants of these substrates, together with the selected crystallographic planes, are presented in Table 2. Additionally, lattice constants corresponding to diagonal growth and 2 × 2 superlattice growth modes were estimated to facilitate comparison with the WO_3_ lattice parameters listed in Table 1. Furthermore, we calculated and reported the lattice mismatches (%) between each WO_3_ phase and each substrate plane, taking into account both the “diagonal” and “2 × 2” epitaxial configurations where applicable. The results are presented in Table S1.

It is noteworthy that the YAO and SLAO substrates possess distinctive characteristics warranting further clarification. YAO exhibits an orthorhombic crystal structure analogous to that of DyScO_3_, NdGaO_3_, or TbScO_3_ [38]. The chosen orientation corresponds to the (110) plane in the orthorhombic system, which is equivalent to the (100) plane in the pseudocubic framework, with a pseudocubic lattice constant of approximately 3.72 Å. Some literature sources refer to this orientation as (100) within the pseudocubic lattice [39]. In the case of SLAO, which crystallizes in a tetragonal system, the (001) plane was selected, analogous to the (100) planes of SrTiO_3_ substrates. Furthermore, this investigation incorporated fused silica substrates, characterized by an amorphous structure, to fabricate WO_3_ thin films. These served as control samples to benchmark against films grown on single-crystal substrates.

### 2.2. Sample Preparation and Characterization

WO_3_ thin films were fabricated via Laser-MBE utilizing a Pioneer 180 system (Necera, Houston, TX, USA) on double-side polished single-crystal substrates as well as fused silica substrates measuring 10 × 10 × 0.5 mm^3^. During deposition, a 248 nm KrF excimer laser was focused onto a high-purity WO_3_ solid target (99.99%, MTI, Hefei, China) within the deposition chamber, delivering an energy density of 90 mJ/mm^2^. This laser irradiation induced rapid localized surface evaporation, generating a plasma plume composed of ablated species. The laser pulse frequency was 5 Hz, and the laser spot size on the target surface was approximately 2.5 mm^2^. The target–substrate distance was maintained at 50 mm. High-purity oxygen (99.999%) was introduced at flow rates of 0.1–100 sccm (corresponding to an oxygen partial pressure of 0.1–100 Pa). The base pressure of the chamber before oxygen introduction was approximately 1 × 10^−5^ Pa. These species subsequently migrated toward the heated substrate surface, facilitating the growth of WO_3_ thin films under 600 °C and 5 Pa oxygen partial pressures. The substrate temperature was monitored using a thermocouple, with a control accuracy of ±2 °C. No post-annealing treatment was applied to the samples. Following deposition, the WO_3_ films were transferred directly to a magnetron sputtering system, where Pd nanoparticles with nominal thicknesses of approximately 3 nm were deposited.

Structural characterization of the as-deposited WO_3_ films was conducted employing a suite of complementary analytical techniques. Crystalline phase identification was performed using X-ray diffraction (XRD) with Cu Kα radiation (λ = 1.5406 Å) on a Bruker D8 Advance diffractometer (Bruker, Billerica, MA, USA). High-resolution transmission electron microscopy (HR-TEM, JEM-2100F, JEOL, Tokyo, Japan), provided nanoscale crystallographic insights. Surface morphology was examined via field-emission scanning electron microscopy (SEM, Gemini 300, ZEISS, Jena, Germany) and atomic force microscopy (AFM, Multimode VIII, Bruker, Billerica, MA, USA) operated in tapping mode. Additionally, the film thickness was determined using a stylus profilometer (Bruker Dektak XT, Billerica, MA, USA) under a tip force of 3 mg on samples prepared with a masked step edge.

### 2.3. Hydrogen Sensing Measurement

The high-precision automated electrical and optical dual-mode gas sensor characterization measurement system, developed by the research team, was utilized to evaluate the performance of the hydrogen sensor [40]. The gas atmosphere used for hydrogen sensing measurements was generated using a vacuum-assisted partial pressure gas-mixing system consisting of two chambers: a gas-mixing chamber (ISO-200, volume 4256 mL) and a test chamber (KF-flange six-port, volume 958 mL). Before each measurement, the mixing chamber was evacuated and subsequently filled sequentially with high-purity H_2_ and synthetic air using mass-flow controllers (accuracy ±1% F.S.). The final hydrogen concentration was defined by the partial pressures of the individual gases, monitored by an absolute-pressure sensor with ±0.25% reading accuracy. After gas preparation, the test chamber was evacuated, and the valve connecting the two chambers was opened. Because the mixed gas was maintained at 1.225 atm, the test chamber reached 1 atm and was fully filled within approximately 1 s (Figure S1). All standard measurements were conducted under dry conditions, and humidity-controlled mixtures were obtained by introducing water vapor with a specified partial pressure into the mixing chamber prior to hydrogen addition.

To enable simultaneous optical and electrical measurements, the six-port test chamber was equipped with high-transmittance quartz flanges on both the top and bottom. A 785 nm semiconductor laser was directed through the top flange and a 3 mm aperture machined at the center of the conventional electrical sample holder, allowing the beam to pass through the WO_3_ film. Transmitted light was collected by a silicon photodiode detector (Hamamatsu S1337, Hamamatsu City, Japan) positioned beneath the bottom flange. Both the incident and detection sides were fitted with bandpass filters to suppress ambient light interference. The use of a 785 nm probe wavelength was motivated by the responsivity characteristics of the S1337 photodiode: although WO_3_ exhibits its strongest hydrogen-induced absorption changes near 1000 nm, operating close to the photodiode’s cutoff (~1100 nm) would reduce responsivity and introduce signal instability. The 785 nm wavelength therefore provides stable, high-signal-to-noise detection while still probing a spectral region in which WO_3_ exhibits clear hydrogen-induced transmittance modulation. Corresponding absorbance spectra before and after hydrogen coloration are provided in Figure S2 of the Supporting Information. Electrical resistance was measured simultaneously using a Keithley 6517B high-resistance meter (Keithley, Cleveland, OH, USA), which supplied a constant voltage and recorded the resulting current.

The sample testing temperature was controlled at 80 °C or 160 °C. For quantitative comparison, the electrical response was calculated as SE = R_air_/R_H2_ and the optical response as SO = T_air_/T_H2_, where R_air_ and T_air_ denote the baseline electrical resistance and transmittance in air, and R_H2_ and T_H2_ represent the stabilized values under a given hydrogen concentration. The response time and recovery time for both electrical and optical signals were determined using the t_90_ criterion, corresponding to the time required to reach 90% of the total change relative to the stabilized state. These values were extracted from repeated measurements, and the associated statistical uncertainties are shown as error bars in corresponding figures.

## 3. Results and Discussion

### 3.1. WO_3_ Film Deposited on Amorphous Quartz Substrate

Gas-sensitive films are conventionally deposited onto amorphous substrates. For instance, microhotplates typically employ composite films composed of silicon dioxide and silicon nitride (SiO_x_/SiN_x_) [29]. To systematically investigate the influence of single-crystal substrates on the structural and hydrogen sensing properties of WO_3_ films, WO_3_ films were initially deposited on amorphous fused quartz substrates (denoted as WO_3_/a-Qz). Subsequent characterization focused on their structural attributes and hydrogen sensing performance. The deposition parameters for the WO_3_/a-Qz samples were kept consistent with those used for single-crystal substrates. At this stage, it is necessary to clarify the thickness of the WO_3_ films, as this parameter serves as a reference for subsequent comparisons with single-crystal substrates. The film thickness was measured using samples intentionally prepared with a masked step edge, achieved by partially covering the substrate with double-side-polished Si wafers during deposition. The step height was then determined using a stylus profilometer, with the reported values representing the average of six measurement points. The WO_3_ film grown on the amorphous fused quartz substrate exhibits an average thickness of approximately 52 nm. In contrast, the films deposited on single-crystal oxide substrates are significantly thinner, with average thicknesses of ~32 nm on YAO, ~28 nm on SLAO, ~23 nm on LAO, and ~20 nm on STO, and the thickness variations across samples of the same substrate type remain within ±2 nm. This systematic reduction in thickness can be attributed to the distinct surface characteristics of the substrates. The amorphous fused quartz substrate contains abundant dangling bonds and structural defects that facilitate adatom adsorption and yield a higher nucleation density, thereby promoting more efficient film growth. Conversely, the atomically ordered surfaces of the single-crystal substrates impose higher interfacial energies and lower nucleation densities, making adatom incorporation more restrictive and ultimately leading to reduced effective deposition rates.

**Figure 2:**
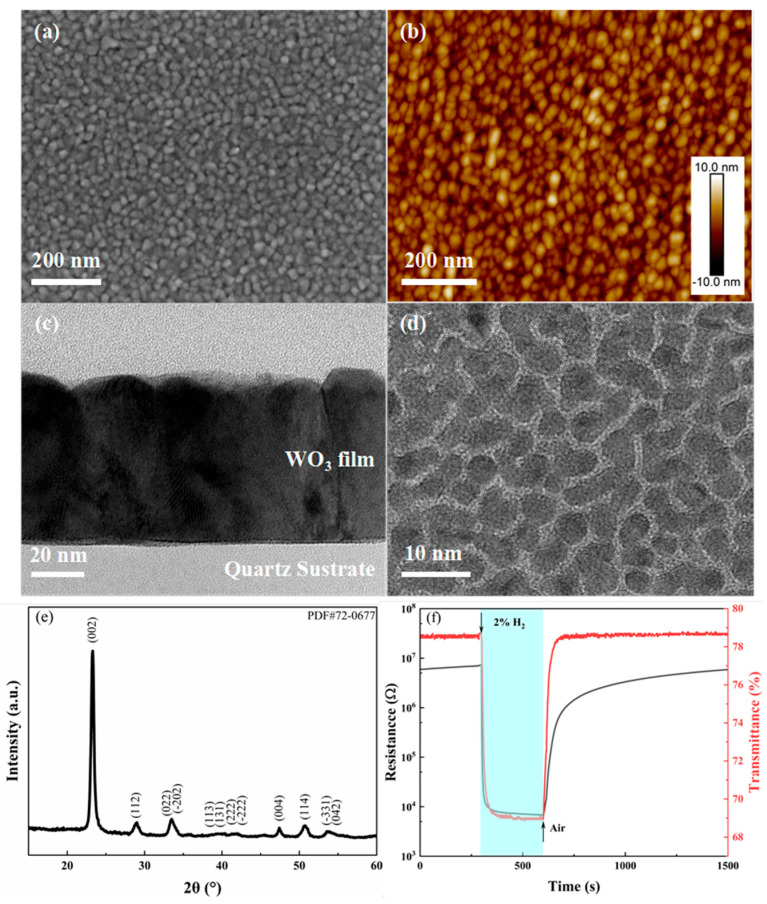
Structure and hydrogen sensing performance of WO_3_/a-Qz sample. (**a**) SEM image, (**b**) AFM image, (**c**) cross-sectional TEM image, (**d**) TEM image of a 3 nm Pd layer deposited on an amorphous carbon TEM grid, (**e**) XRD pattern of the WO_3_/a-Qz sample, and (**f**) electrical and optical responses of the 3 nm Pd catalyzed WO_3_/a-Qz sample to 2% H_2_ in air at 80 °C.

Figure 2a,b illustrates SEM and AFM images of the WO_3_/a-Qz film. The surface morphology reveals a granular texture with relatively uniform grain sizes predominantly ranging from 20 to 30 nm, averaging approximately 26 nm, and a root mean square (RMS) surface roughness of 2.1 nm. Figure 2c presents a cross-sectional transmission electron microscopy (TEM) image of the WO_3_/a-Qz film, which distinctly displays vertically oriented columnar grains extending throughout the entire film thickness. The grain dimensions observed via TEM corroborate the SEM and AFM findings, falling within the 20 to 30 nm range. Furthermore, these columnar grains demonstrate a high degree of alignment in their growth orientation, which is reflected in the XRD pattern (Figure 2e) exhibiting a pronounced preferred orientation. Comparison with standard XRD reference data identifies the film as corresponding to the monoclinic phase of WO_3_ commonly observed at ambient temperature (PDF #72-0677), characterized by the space group P2_1_/n (14). The most intense diffraction peak is attributed to the (002) crystallographic plane. The crystallite size was estimated from the (002) diffraction peak using the Scherrer equation. Instrumental broadening was corrected by measuring a stress-free Si standard under identical conditions. A shape factor of K = 0.9 was chosen because it is commonly used for oxide thin films with approximately spherical to slightly anisotropic crystallites. The resulting coherent domain size is 21 nm. This value is slightly smaller than the 20–30 nm grain sizes obtained from SEM, AFM, and TEM. The difference is expected because Scherrer analysis reflects the size of X-ray coherent scattering domains, whereas SEM/AFM/TEM measure the physical grain size, which can be larger when individual grains contain multiple coherent domains. Overall, the values are consistent and fall within the same characteristic nanoscale regime.

Figure 2d presents a TEM image of 3 nm Pd deposited on a smooth amorphous carbon TEM grid. The image reveals that Pd deposited in high-purity argon at room temperature undergoes typical island growth, with each isolated nanoscale island constituting a single crystal. When Pd is deposited onto the rough surface of the WO_3_ film, the connectivity between Pd islands is further diminished, which does not significantly influence the overall electrical resistance of the film. Figure 2f illustrates the electrical and optical responses of the Pd catalyzed WO_3_/a-Qz sample when exposed to a 2% H_2_/air mixture at 80 °C. The selection of 80 °C was primarily motivated by the aim to enhance the selectivity of the sample and to minimize the overall power consumption of the device [41]. Owing to Pd’s distinctive catalytic properties toward hydrogen, the WO_3_/a-Qz samples demonstrate a pronounced response to hydrogen at 80 °C, while exhibiting minimal responses to other gases. Furthermore, operating at this temperature mitigates interference from ambient temperature fluctuations and humidity. In terms of electrical resistance, the sample exhibits a baseline resistance of 6.9 × 10^6^ Ω in air. Upon H_2_ exposure, the resistance rapidly decreases to approximately 1 × 10^4^ Ω and eventually stabilizes at 6.8 × 10^3^ Ω. This corresponds to a sensor response magnitude of roughly three orders of magnitude, with response and recovery times of 1 s and 770 s, respectively. Concurrently, the optical response is also substantial. The transmittance of pure WO_3_ without Pd modification is approximately 92% at 785 nm, which decreases to 78.6% after Pd coating. Exposure to 2% H_2_ further reduces the optical transmittance to 68.9%, yielding a response magnitude of 9.7%, with response and recovery times of 28 s and 43 s, respectively.

Notably, the optical response time is considerably longer than the electrical response time, whereas the optical recovery time is significantly shorter. This discrepancy arises because the electrical response of WO_3_ to hydrogen involves both surface and bulk reactions, whereas the optical response is predominantly governed by bulk reactions. The surface reaction entails hydrogen interacting with adsorbed oxygen ions (O_2_^−^, O^−^, O^2−^) on the WO_3_ surface [42,43], while the bulk reaction involves the dual injection of hydrogen species, resulting in the formation of tungsten bronze [44,45]. Since the catalytic activity is confined to the WO_3_ surface, the surface reaction proceeds relatively rapidly, whereas the bulk reaction is slower due to the diffusion of injected hydrogen species from the surface into the bulk. Consequently, the injection of hydrogen into the WO_3_ bulk accounts for the extended optical response time. During recovery, the test temperature of 80 °C prolongs the time required for the WO_3_ surface to be re-adsorbed by oxygen ions and re-establish equilibrium. Having elucidated the structural characteristics of polycrystalline WO_3_ films prepared on amorphous substrates and their corresponding optical and electrical responses to hydrogen, the subsequent focus shifts to WO_3_ films grown on single-crystal substrates. Their hydrogen response characteristics will be examined and compared with those of the polycrystalline films.

### 3.2. WO_3_ Film Deposited on Single Crystal Substrates

**Figure 3:**
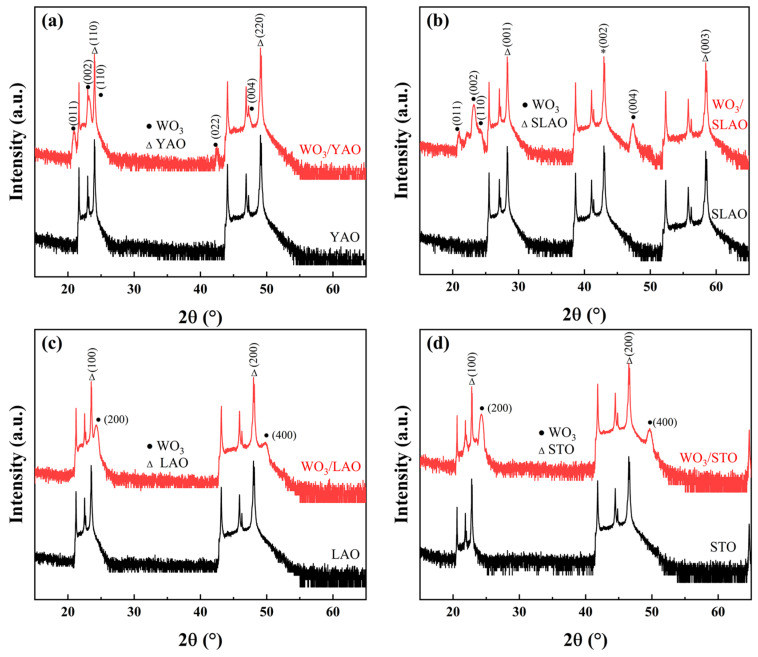
X-ray diffraction patterns of WO_3_ films deposited on different single-crystal substrates: (**a**) WO_3_/YAO, (**b**) WO_3_/SLAO, (**c**) WO_3_/LAO, (**d**) WO_3_/STO.

XRD analysis was initially conducted on samples deposited onto oxide single-crystal substrates to identify their crystal phases and the exposed crystallographic planes. The measurements employed a Bragg–Brentano (B-B) focusing geometry. Due to the exceptionally high diffraction intensities observed in the single-crystal substrates, a 0.2 mm nickel filter was utilized as an attenuator to prevent detector saturation. The resulting diffraction patterns are presented in Figure 3, where the principal diffraction peaks corresponding to WO_3_ and the four substrate materials are annotated. Given the substantial variation in peak intensities, the data are represented on a logarithmic scale for clarity.

Comparison with powder diffraction reference cards from the Inorganic Crystal Structure Database (ICSD) revealed that WO_3_ films grown on YAlO_3_ (YAO) and SrLaAlO_4_ (SLAO) substrates crystallize in the monoclinic phase, consistent with PDF #87-2380, space group Pc (7). Conversely, WO_3_ deposited on LaAlO_3_ (LAO) and SrTiO_3_ (STO) substrates adopts an orthorhombic structure, corresponding to PDF #71-0131, space group Pmnb (62). Although the orthorhombic phase is often described in the literature using the Pbcn setting, this phase is crystallographically equivalent to the Pmnb setting adopted here; the difference arises solely from axis permutation conventions. All peak indices in this work follow the Pmnb setting for consistency with the ICSD reference. Examination of the lattice parameters listed in Table 1 and Table 2 and Supplementary Table S1 indicates that WO_3_ preferentially grows on single-crystal substrates in a manner that minimizes lattice mismatch. Although substrate-induced stress plays a central role, the resulting strain does not follow a simple monotonic trend with lattice mismatch, because WO_3_ minimizes its elastic energy by adopting different crystal phases and growth configurations on different substrates. As a result, the measured strain values of the films fall within a narrow range and do not exhibit a one-to-one correspondence with the sensing response, making strain an unsuitable horizontal axis for representing the combined stress–phase–microstructure effects.

Furthermore, the WO_3_ films on YAO and SLAO exhibit three prominent diffraction peaks between 20° and 30°, attributable to the (110), (002), and (011) crystallographic planes, respectively. Notably, the intensity of the (002) peak exceeds that of the other two by more than a factor of 15, signifying a pronounced preferred orientation during film growth. In contrast, the relatively larger lattice constants of the LAO and STO substrates induce interfacial strain, which leads to distortion and tilting of the WO_6_ octahedra within the WO_3_ lattice [31,36]. This structural modification enhances the crystal symmetry, stabilizing the orthorhombic phase that is typically observed within the temperature range of 350 °C to 720 °C, consistent with the deposition temperature of 600 °C employed in this study. Additionally, WO_3_ films grown on LAO and STO substrates display diffraction peaks exclusively corresponding to the (200) and (400) planes, with the absence of other crystallographic reflections. Although θ–2θ scans confirm strong out-of-plane preferred orientation and allow identification of the dominant diffraction planes, they cannot fully rule out the presence of partial fiber texture. Therefore, the structural discussion refers to the dominant out-of-plane orientation rather than a complete epitaxial relationship. Nevertheless, the out-of-plane lattice matching between the WO_3_ film and the underlying substrate is sufficient to impose interfacial stress, which can modify the WO_6_ octahedral tilting and stabilize the orthorhombic phase even in the presence of fiber-textured growth. Thus, the key conclusions regarding stress-induced phase modulation remain valid.

**Figure 4:**
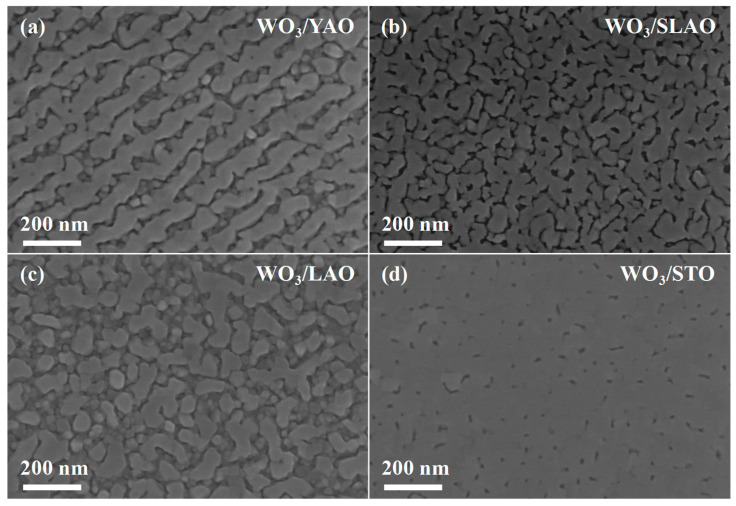
Scanning electron microscope images of WO_3_ films deposited on different single-crystal substrates: (**a**) WO_3_/YAO, (**b**) WO_3_/SLAO, (**c**) WO_3_/LAO, (**d**) WO_3_/STO.

**Figure 5:**
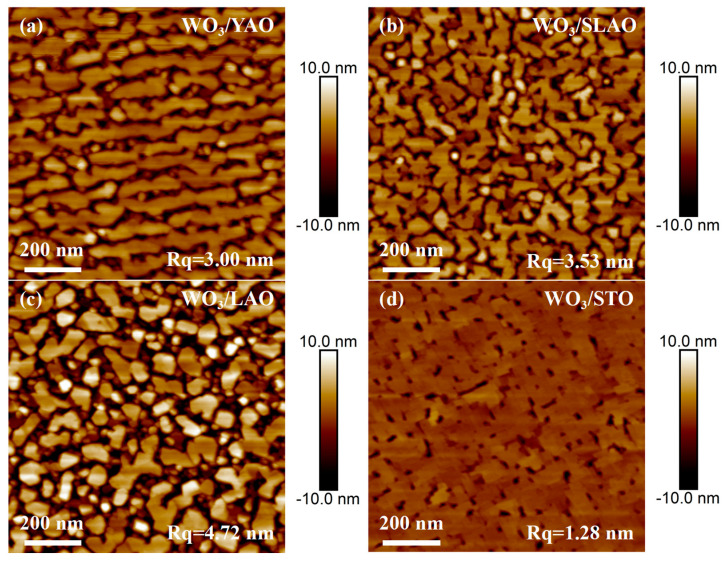
Atomic force microscopy images of WO_3_ films deposited on different single-crystal substrates: (**a**) WO_3_/YAO, (**b**) WO_3_/SLAO, (**c**) WO_3_/LAO, (**d**) WO_3_/STO.

Figure 4 presents SEM images of four WO_3_ samples. In contrast to the granular surface morphology observed in WO_3_ films deposited on amorphous quartz substrates, the WO_3_ films grown on YAO and LAO substrates predominantly exhibit smooth surface regions; nevertheless, granular WO_3_ structures are present within the interstitial spaces between these smooth areas. The extent of smooth surface coverage further increases for films grown on SLAO and STO substrates, with the WO_3_/SLAO sample displaying an almost complete absence of granules, and the WO_3_/STO sample exhibiting a surface nearly entirely covered by a flat film. It is noteworthy that WO_3_ films grown on YAO and SLAO substrates adopt a monoclinic phase, whereas those grown on LAO and STO substrates crystallize in an orthorhombic phase. Given that the lattice mismatch associated with SLAO and STO substrates is greater than that of YAO and LAO substrates (refer to Table 1 and Table 2), it can be inferred that an increase in lattice mismatch promotes a more layered growth mode in WO_3_ films. Figure 5 shows AFM images of the same four samples, corroborating the SEM observations and further substantiating the tendency of WO_3_ films with different crystal phases to adopt layered morphologies as lattice mismatch increases. Among the samples analyzed, the WO_3_/STO film exhibits the lowest surface roughness, measured at 1.28 nm.

### 3.3. Hydrogen Sensing Properties

Prior to performing hydrogen sensitivity assessments, a Pd nanoparticle layer with a nominal thickness of 3 nm was deposited onto the sample surfaces via magnetron sputtering to serve as a catalyst for hydrogen detection. Subsequently, all samples were subjected to an aging process for more than 24 h under alternating cycles of 2% H_2_/air and pure air to establish a stable response state. The differences in electrical and optical behavior before and after aging are presented in Figure S3.

**Figure 6:**
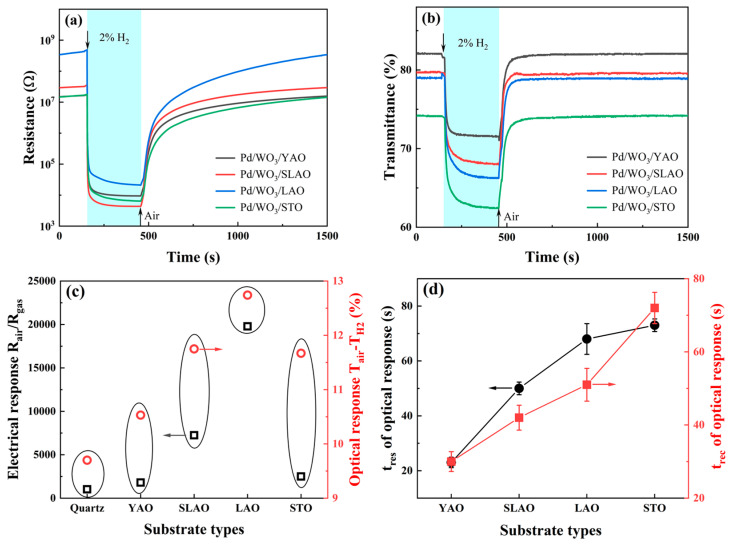
Response curves of WO_3_ grown on four single-crystal substrates to 2 vol.% hydrogen at an operating temperature of 80 °C: (**a**) resistance response; (**b**) transmittance response; (**c**) response magnitude; (**d**) optical response and recovery times.

Initially, the H_2_ sensing capabilities of WO_3_ films grown on four distinct single-crystal substrates were evaluated at an operating temperature of 80 °C. The optical and electrical response profiles, along with corresponding response magnitudes and response times, are presented in Figure 6. Relative to the polycrystalline WO_3_ sample deposited on a fused quartz substrate (Figure 2f), all four single-crystal substrate samples demonstrated marked enhancements in both electrical and optical responses. Notably, the response magnitude exhibited a non-monotonic trend, increasing initially and subsequently decreasing as the lattice constant expanded (Figure 6c). The polycrystalline WO_3_/a-Qz sample exhibited an electrical resistance response magnitude of 1022 and an optical transmittance variation of 9.7%, with electrical and optical response times of 1 s and 28 s, respectively. Among the single-crystal substrate samples, the WO_3_/YAO specimen displayed the lowest response magnitude, with an electrical response magnitude of 1730 and an optical transmittance change of 10.5%. Conversely, the WO_3_/LAO sample exhibited the highest response magnitude, achieving an electrical response magnitude of 19,702 and an optical transmittance change of 12.7%. The superior H_2_ sensing performance of the WO_3_/LAO sample is primarily attributed to interfacial stress induced by the LAO substrate, which facilitates a phase transition in the WO_3_ thin film from the conventional monoclinic phase to a high-temperature orthorhombic phase [34]. Furthermore, the exposed crystal plane shifted from (002) to (200), while the WO_3_/LAO sample maintained favorable surface roughness. In contrast, the WO_3_/STO sample, despite sharing the same crystal structure and exposed crystal plane, exhibited a layered growth mode that significantly diminished surface roughness. Following Pd deposition, both the electrical resistance and optical transmittance of the samples in air decreased substantially, resulting in a suppression of the response magnitude. This interpretation focuses on the out-of-plane structural evolution detectable from θ–2θ diffraction, which is the component most strongly coupled to substrate-induced interfacial stress.

However, the enhanced crystallinity of samples grown on single-crystal substrates introduced certain drawbacks, notably a pronounced deceleration in response and recovery kinetics, particularly evident in the optical response data associated with bulk reactions. As depicted in Figure 6d, the optical response time progressively increased from 23 s for the WO_3_/YAO sample to 73 s for the WO_3_/STO sample, while the recovery time extended from 30 s to 72 s. This retardation in optical response and recovery is primarily ascribed to the increased density of the samples due to improved crystallinity, which reduces gas diffusion pathways between the columnar grains present in the WO_3_/a-Qz sample. Additionally, phase transitions observed in the WO_3_/LAO and WO3/STO samples further impede hydrogen ingress and egress. The slow optical response observed at 80 °C can be rationalized as a diffusion-limited process. Previous studies have reported activation energies of approximately 0.24–0.43 eV for hydrogen-related diffusion in WO_3_ [36,46], which is consistent with the strong temperature dependence observed here. This agreement suggests that thermally activated hydrogen migration plays a dominant role in governing optical kinetics.

Concerning the electrical response, which encompasses both surface and bulk reactions, the response times across all four single-crystal substrate samples remained approximately 1 s, comparable to that of the WO_3_/a-Qz sample characterized by a polycrystalline columnar crystal structure. Nevertheless, the recovery times were substantially prolonged. The WO_3_/a-Qz sample exhibited a recovery time of 770 s, whereas none of the four single-crystal substrate samples achieved full recovery within the 30 min testing interval. This phenomenon is primarily attributed to the difficulty in extraction of hydrogen species that have penetrated the interior of the single-crystal substrate samples, resulting in a fraction of hydrogen being irreversibly trapped within the WO_3_ films and thereby hindering complete electrical response recovery over short durations. Using the WO_3_/LAO sample as a representative case, we further conducted cross-selectivity and humidity-dependence tests. As shown in Figures S4 and S5, both the electrical and optical signals exhibit excellent selectivity toward H_2_ at 80 °C, with other common interfering gases such as CO, CH_4_, NH_3_, and H_2_S producing only minimal responses. The influence of humidity manifests primarily through the competitive occupation of adsorption sites. Water molecules, adsorbing on the WO_3_ surface in either molecular or hydroxyl form, reduce the availability of reactive oxygen species, leading to a concurrent decrease in baseline resistance and in the overall H_2_ response magnitude.

**Figure 7:**
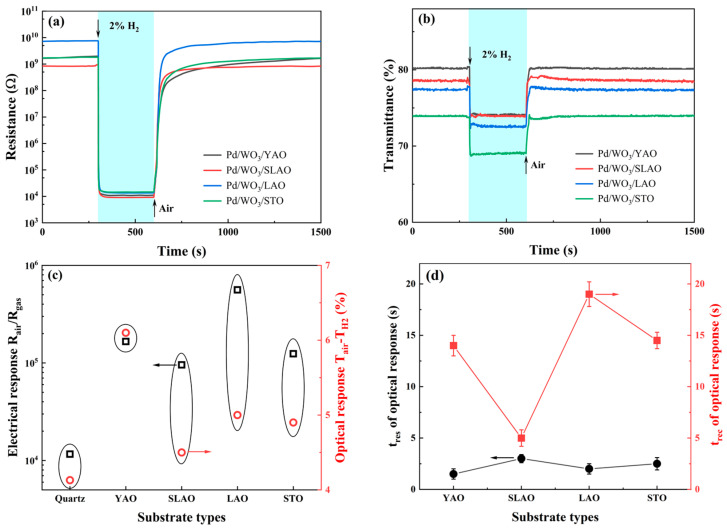
Response curves of WO_3_ grown on four single-crystal substrates to 2 vol.% hydrogen at an operating temperature of 160 °C: (**a**) resistance response; (**b**) transmittance response; (**c**) response magnitude (**d**) optical response and recovery times.

To address the challenge of recovering WO_3_ thin films deposited on single-crystal substrates and to assess their practical applicability, the operating temperature was elevated from 80 °C to 160 °C, followed by evaluation of their response to 2% H_2_ gas. As illustrated in Figure 7, this temperature increases markedly enhanced both the electrical and optical response speeds of the samples, with the electrical response achieving full recovery. Notably, the amplitude changes in the optical and electrical responses exhibited inverse trends. Specifically, the optical response amplitude diminished by approximately 50% (Figure 7c), decreasing from a range of 10.5–12.7% at 80 °C to 4.9–6.1% at 160 °C. Similarly, the WO_3_/a-Qz sample’s optical response amplitude declined from 9.7% to 4.1%. A comparison between Figure 6b and Figure 7b reveals that the transmittance in air (T_air_) remained nearly constant across both temperatures, whereas the equilibrium transmittance in H_2_ (T_H2_) increased significantly at 160 °C, thereby reducing the optical response amplitude. This behavior is consistent with the thermally activated extraction of hydrogen from the WO_3_ lattice and the accelerated re-adsorption of oxygen species at elevated temperature, which reduces the equilibrium hydrogen concentration and thereby suppress the optical contrast.

The optical response, characteristic of a bulk phenomenon, is directly correlated with the hydrogen species concentration within WO_3_ film at reaction equilibrium. This equilibrium is governed by the dynamic interplay between hydrogen species injection and extraction, the latter facilitated by reaction with environmental oxygen. Evidently, elevated operating temperatures accelerate hydrogen extraction rates under oxygen influence, shifting the equilibrium toward a lower hydrogen content within WO_3_. Consequently, T_H2_ increases at higher temperatures, leading to a diminished optical response amplitude. Furthermore, both the response and recovery times of the optical response were substantially shortened at 160 °C. At 80 °C, these times typically ranged from 23 to 73 s for WO_3_ samples on single-crystal substrates; upon increasing the temperature, response times decreased to 1.5–3 s and recovery times to 5–20 s, thereby significantly enhancing sample practicality. At 80 °C, the WO_3_/LAO films exhibited an irreversible fraction of approximately 8–10%, which can be attributed to the limited mobility of hydrogen-related species within the WO_3_ lattice and their partial retention after exposure, as well as the slow re-adsorption and saturation of chemisorbed oxygen on the surface at this temperature. In contrast, at 160 °C, the films displayed fully reversible electrical and optical responses over multiple cycles (Figure S6), confirming that dehydrogenation of the sample and subsequent re-adsorption of oxygen are thermally activated and readily repeatable.

Regarding the electrical response, all WO_3_ samples on single-crystal substrates, including WO_3_/a-Qz, demonstrated an increase ranging from tenfold to one hundredfold in response amplitude at 160 °C. The WO_3_/YAO sample demonstrated the most pronounced enhancement, with its electrical response amplitude rising from 1730 at 80 °C to 165,990. Moreover, the WO_3_/YAO sample maintained the highest electrical response amplitude of 560,720, representing a 28-fold increase relative to 80 °C. Analysis of Figure 6a and Figure 7a indicates that this amplification primarily results from a substantial increase in resistance in air (R_air_) at 160 °C. This phenomenon is attributed to the promotion of chemical oxygen adsorption at elevated temperatures, which thickens the depletion layer and consequently elevates R_air_.

The WO_3_/LAO sample exhibited superior electrical response compared to other samples, a performance partially attributable to its higher surface roughness. As a result, following the deposition of palladium at an equivalent thickness, the sample exhibited the highest resistance in air, which corresponded to an increased response amplitude. An additional contributing factor could be the presence of the high-energy orthorhombic phase, which appears to be more favorable for hydrogen adsorption reactions on the surface. Notably, the high-temperature orthorhombic phase exposes the atypical (200) crystal plane. Although a direct comparison of surface energies cannot be made without DFT calculations, previous reports suggest that this facet exhibits a mixed W-O termination and a relatively lower density of surface W atoms compared to the (020) plane, enabling more accessible oxygen chemisorption and higher surface reactivity [47]. Therefore, the (200) plane is likely to possess enhanced surface activity, which may contribute to the improved sensing performance observed in this study.

Although the STO sample also possesses an orthorhombic structure, its surface roughness is considerably lower than that of other samples. As a result, following surface modification with 3 nm Pd, both its resistance and transmittance in air were relatively low, leading to weaker electrical and optical response amplitudes. Additionally, at 160 °C, the response time of the samples decreased from 1 s at 80 °C to 0.5 s, which is significantly faster than those reported for most WO_3_-based hydrogen sensors [14,15,16,17,18,19,20,21], thereby satisfying the requirements for single-use leak-alarm applications. Although fabricating thinner WO_3_ films or patterned porous structures on LAO and STO substrates would help further decouple the effects of phase stabilization and gas diffusion pathways, producing ultrathin WO_3_ layers on these substrates would result in extremely high electrical resistance beyond our measurable range. Moreover, implementing controlled porosity requires additional process development. Therefore, these directions will be considered in future work.

## 4. Conclusions

In summary, interfacial stress imposed by single-crystal oxide substrates is shown to be a key factor governing the structural and functional characteristics of WO_3_ hydrogen sensors. The films adopt either monoclinic (YAO, SLAO) or orthorhombic (LAO, STO) phases depending on the strain-minimization pathway, and this phase selection exerts a far stronger influence on hydrogen sensitivity than the residual strain itself. The orthorhombic WO_3_/LAO film, in particular, demonstrates markedly superior performance, achieving an electrical response of 1.97 × 10^4^, an optical transmittance changed of 12.7%, and an electrical response time of 1 s at 80 °C toward 2% H_2_. At an elevated temperature of 160 °C, the response time further decreases to 0.5 s, and the recovery time shortens to approximately 20 s, meeting the operational requirements for real-time hydrogen leak alarm applications. It should be emphasized that the enhanced practicality demonstrated here refers specifically to applications requiring fast, reversible hydrogen sensing, and arises from the combined effects of stress-induced phase stabilization, film texture, and gas diffusion pathways rather than from a monotonic strain–response relation. These insights highlight the potential of interfacial-stress engineering as a powerful strategy for designing high-performance WO_3_-based hydrogen sensors.

## Figures and Tables

**Table 1:** Crystalline phases and lattice parameters of WO_3_.

Material	Crystalline Phase	Lattice Parameter						ICSD PDF No.
		*a* (Å)	*b* (Å)	*c* (Å)	*α* (°)	*β* (°)	*γ* (°)	
WO_3_	Triclinic	7.309	7.522	7.678	88.81	90.92	90.93	71-0305
WO_3_	Monoclinic	5.277	5.156	7.663	90	91.76	90	87-2380
WO_3_	Monoclinic	7.306	7.540	7.692	90	90.88	90	72-0677
WO_3_	Orthorhombic	7.333	7.573	7.740	90	90	90	89-4477
WO_3_	Orthorhombic	7.341	7.570	7.754	90	90	90	71-0131
WO_3_	Tetragonal	5.250	5.250	3.915	90	90	90	85-0808
WO_3_	Hexagonal	7.324	7.324	7.662	90	90	120	85-2460

**Table 2:** Information of selected single crystal substrates used for WO_3_ deposition.

Substrate	Crystalline Phase	ICSD PDF No.	Lattice Constant			Crystal Plane Used	Diagonal (Å)	“2 × 2” Superlattice (Å)
			*a* (Å)	*b* (Å)	*c* (Å)			
YAlO_3_	Orthorhombic	89-7947	5.179	5.327	7.37	(110)	5.254	7.430
SrLaAlO_4_	Tetragonal	81-0744	3.756	3.756	12.636	(001)	5.311	7.512
LaAlO_3_	Cubic	70-4125	3.82	3.82	3.82	(100)	5.401	7.640
SrTiO_3_	Cubic	79-0175	3.905	3.905	3.905	(100)	5.522	7.810

## Data Availability

All data are available from the corresponding author upon request.

## Supplementary Materials

The following supporting information can be downloaded at: https://www.mdpi.com/article/10.3390/nano15231785/s1, Figure S1. Pressure evolution in the test chamber during gas switching. Figure S2. Absorbance spectra of Pd/WO_3_ measured in air and 2% H_2_/air mixture. Figure S3. (a) Optical and (b) electrical response curves of WO_3_ films before and after the aging process. Figure S4. (a) Electrical and (b) optical responses of the WO_3_/LAO film to various interfering gases. Figure S5. Electrical and optical responses of WO_3_/LAO under different humidity levels. Figure S6. Multi-cycle electrical response of WO_3_/LAO at 80 °C and 160 °C, showing reversible behavior at elevated temperature. Table S1. Lattice mismatches for the possible epitaxial relationships between different WO_3_ phases and the single-crystal oxide substrates.
