# Supplementary material for: Influence of Interfacial Stress on the Structural Characteristics and Hydrogen Sensing Performance of WO3 Films
Source: Nanomaterials (Basel). 2025 Nov 27;15(23):1785. doi: 10.3390/nano15231785 (PMC12693085; doi:10.3390/nano15231785)
Supplement: Supplementary file 1 [file nanomaterials-15-01785-s001.zip › nanomaterials-3961223-supplementary.pdf]

Supporting information

Zhihong Qiao[1], Jianmin Ye[1], Wen Ye[1*], Jie Wei[2], Ying Li[3], Zhe Lv[4], and Meng Zhao[1*]

[1] School of Physical Science and Technology, Suzhou University of Science and Technology, Suzhou 215000, China

[2] School of Information and Communication, Harbin Institute of Technology, Harbin 150001, China

[3] Advanced Microscopy and Instrumentation Research Center, Harbin Institute of Technology, Harbin 150080, China

[4] School of Electronic and Information Engineering, Suzhou University of Science and Technology, Suzhou 215000, China

* Correspondence: yewen@usts.edu.cn (W.Y.); mzhao@usts.edu.cn (M.Z.)

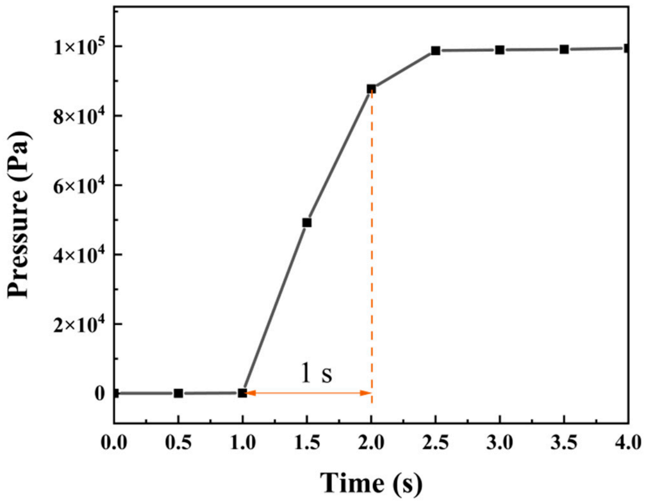

Figure S1. Pressure evolution in the test chamber during gas switching.

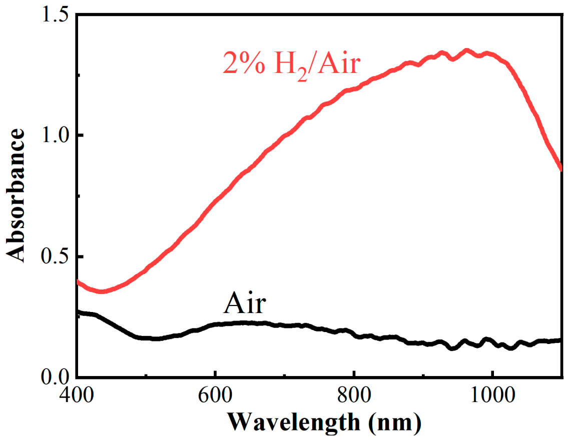

Figure S2. Absorbance spectra of Pd/WO_3 measured in air and 2 % H_2/air mixture.

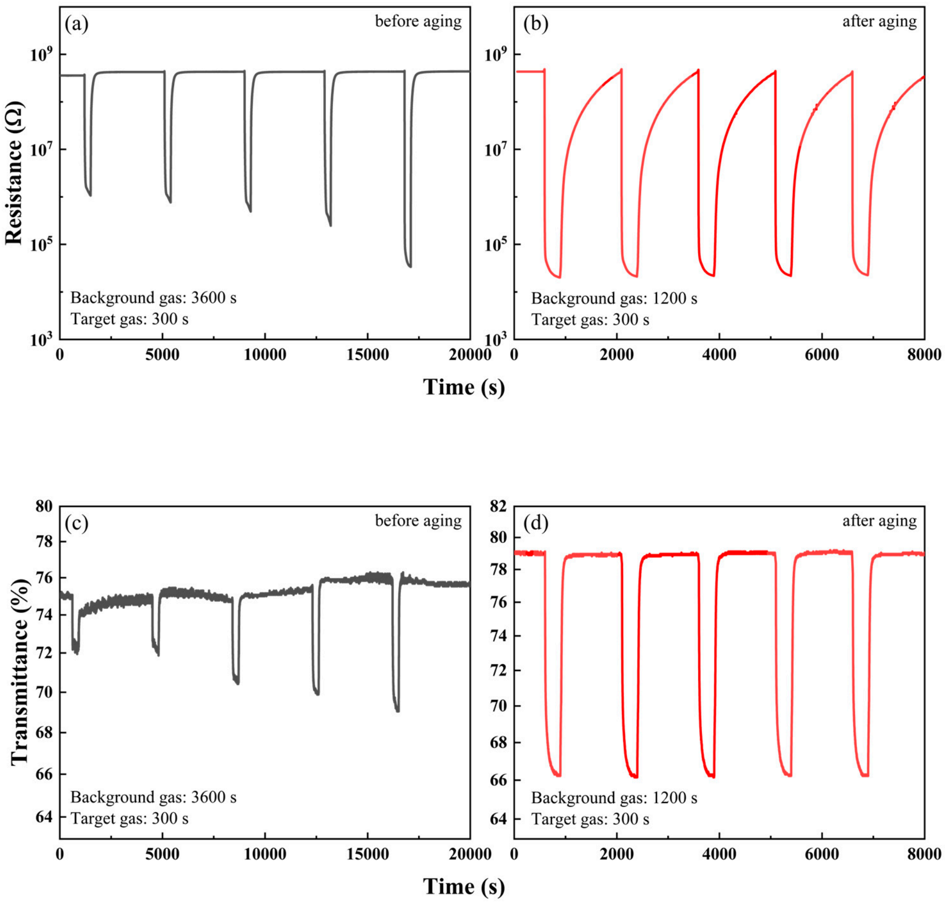

Figure S3. (a) Optical and (b) electrical response curves of WO_3 films before and after the aging process.

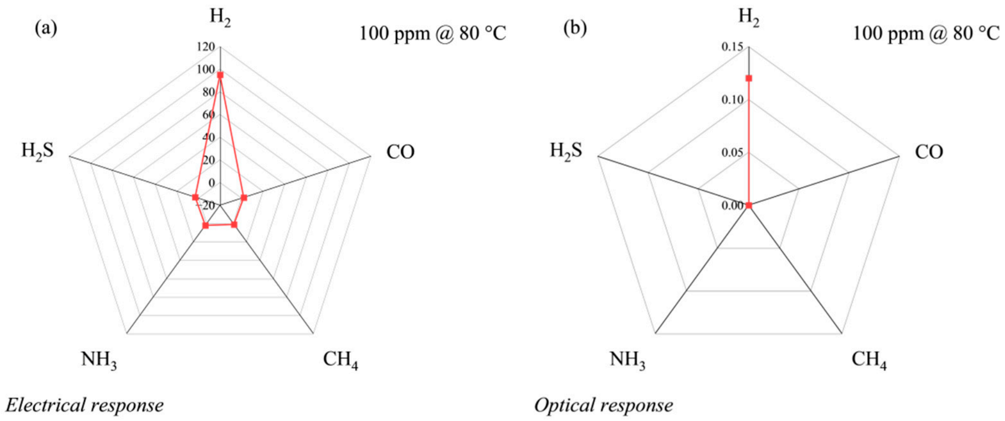

Figure S4. (a) Electrical and (b) optical responses of the WO_3/LAO film to various interfering gases.

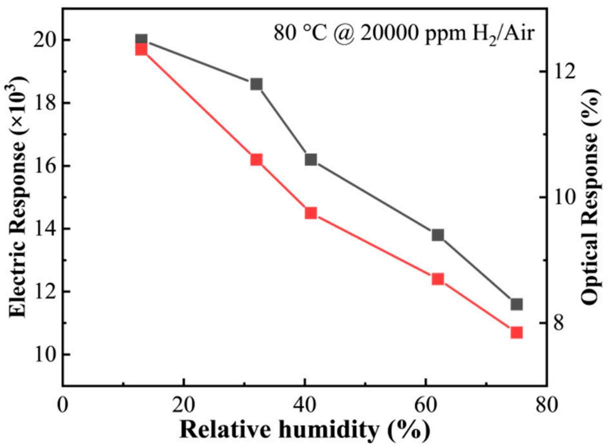

Figure S5. Electrical and optical responses of WO_3/LAO under different humidity levels.

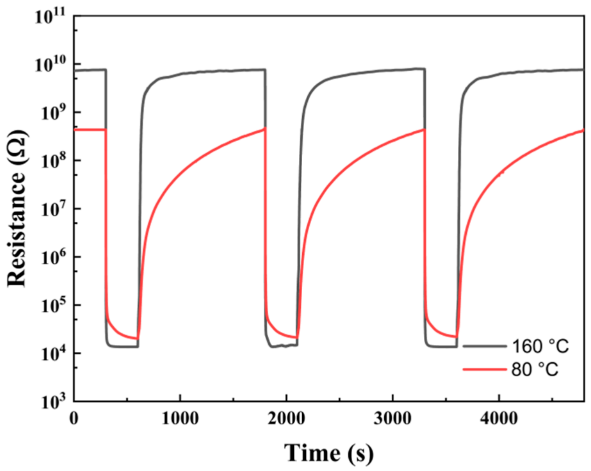

Figure S6. Multi-cycle electrical response of WO_3/LAO at 80 °C and 160 °C, showing reversible behavior at elevated temperature.

Table S1. Lattice mismatches for the possible epitaxial relationships between different WO_3 phases and the single-crystal oxide substrates

Material	Crystal System	Crystal Orientation	$YAlO_3$		$SrLaAlO_4$		$LaAlO_3$		$SrTiO_3$	
			Diagonal	"2x2"	Diagonal	"2x2"	Diagonal	"2x2"	Diagonal	"2x2"
WO_3	Triclinic	(100)	/	-0.19 %	/	-1.29 %	/	-3.02 %	/	-5.31 %
		(001)	/	+2.24 %	/	+1.16 %	/	-0.53 %	/	-2.76 %
WO_3	Monoclinic	(100)	-0.71 %	/	-1.80 %	/	-3.53%	/	-5.85%	/
		(001)	/	/	/	/	/	/	/	/
WO_3	Monoclinic	(100)	/	-0.09 %	/	-1.20 %	/	-2.92 %	/	-5.21 %
		(001)	/	+2.44 %	/	+1.37 %	/	-0.32 %	/	-2.55 %
WO_3	Orthorhombic	(100)	/	+0.31 %	/	-0.79 %	/	-2.51 %	/	-4.79 %
		(001)	/	+2.96 %	/	+1.89 %	/	+0.22 %	/	-2.0 %
WO_3	Orthorhombic	(100)	/	+0.35 %	/	-0.75 %	/	-2.47 %	/	-4.75 %
		(001)	/	+3.03 %	/	+1.96 %	/	+0.29 %	/	-1.93 %
WO_3	Tetragonal	(100)	-0.08 %	/	-1.16 %	/	-2.88%	/	-5.18%	/
		(001)	/	/	/	/	/	/	/	/
WO_3	Hexagonal	(100)	/	-1.45 %	/	-2.57 %	/	-4.31 %	/	-6.64 %
		(001)	/	+0.84 %	/	-0.25 %	/	-1.96 %	/	-4.23 %

Notes:

1. Values highlighted in red indicate the phase and out-of-plane orientation that were experimentally observed for each WO_3 film.

2. For the films grown on $YAlO_3$ and $SrLaAlO_4$, additional weak reflections corresponding to (110) or (011) orientations were detected in the XRD patterns, suggesting minor orientation variants associated with strain-relaxation pathways.

3. The films grown on $LaAlO_3$ and $SrTiO_3$ exhibit strong out-of-plane orientation consistent with stress-stabilized growth, although θ–2θ measurements cannot fully distinguish between highly textured and fully epitaxial growth modes.
